# Patients’ Experiences of a Nurse-Led, Home-Based Heart Failure Self-management Program: Findings From a Qualitative Process Evaluation

**DOI:** 10.2196/28216

**Published:** 2021-04-27

**Authors:** Ying Jiang, Karen Wei Ling Koh, Hadassah Joann Ramachandran, Yee Kian Tay, Vivien Xi Wu, Shefaly Shorey, Wenru Wang

**Affiliations:** 1 Alice Lee Centre for Nursing Studies Yong Loo Lin School of Medicine National University of Singapore Singapore Singapore; 2 National University Heart Centre Singapore National University Hospital Singapore Singapore; 3 Regional Health System National University Health System Singapore Singapore

**Keywords:** self-care, psychosocial educational, nurse-led, mHealth, self-management, heart failure, process evaluation, nursing, mobile phone

## Abstract

**Background:**

Heart failure (HF) is a major public health problem that places a significant disease burden on society. Self-care is important in the management of HF because it averts disease progression and reduces the number of hospitalizations. Effective nursing interventions promote HF self-care.

**Objective:**

This study aims to explore participants’ perspectives on a nurse-led, home-based heart failure self-management program (HOM-HEMP) in a randomized controlled trial conducted in Singapore to gain insight into the effectiveness of the study intervention.

**Methods:**

A descriptive, qualitative approach was used. English- or Chinese-speaking participants from the intervention arms were recruited through a purposive sampling method from January 2019 to July 2019. Individual, face-to-face, semistructured interviews were conducted with 11 participants. All interviews were audio recorded and transcribed verbatim, with the participant identifiers omitted to ensure confidentiality. The thematic analysis approach was used to identify, analyze, and report patterns (themes) within the data.

**Results:**

A total of six themes emerged from the process evaluation interviews and were categorized according to the Donabedian structure-process-outcome framework as *intervention structure*, *intervention process*, and *intervention outcome*. These six themes were manageability of the intervention, areas for improvement, benefits of visiting, personal accountability in self-care, empowered with knowledge and skills in self-care after the intervention, and increased self-efficacy in cardiac care.

**Conclusions:**

The findings of the process evaluation provided additional information on participants’ perceptions and experiences with the HOM-HEMP intervention. Although a home visit may be perceived as resource intensive, it remains to be the preferred way of engagement for most patients. Nurses play an important role in promoting HF self-care. The process of interaction with the patient can be an important process for empowering self-care behavior changes.

## Introduction

### Background

Heart failure (HF) is a major public health problem that places a significant disease burden on society. It is one of the most common causes of hospital admissions and readmissions, leading to an increased demand for hospital beds, staff resources, subsidies, and insurance coverage [1]. Southeast Asians are at particular risk of HF because of the high prevalence of risk factors such as hypertension and diabetes. They also have an earlier onset of HF than Americans and Europeans [2]. Furthermore, the incidence and prevalence of HF are closely related to age. As one gets older, the condition becomes more common [3]. Singapore has one of the fastest aging populations among developed countries worldwide. Therefore, it is expected that the number of people with HF is likely to surge in the near future.

To sustain the health care system, the Ministry of Health in Singapore recently announced three paradigm shifts: beyond hospital to community, beyond quality to value, and beyond health care to health [4]. With this change, more patients with chronic conditions will be managed in the community, and nurses will need to embrace new knowledge and skills that match the care needs of their patients. Simultaneously, as hospital stays become shorter and less frequent, there is a need for patients and families to acquire disease self-care skills to achieve the success of managing their conditions outside the hospital.

In HF, self-care encompasses a range of behaviors, including adhering to medication and treatment, avoiding excessive fluid and salt intake, monitoring daily weight, engaging in exercise, monitoring and identifying exacerbating symptoms, and taking appropriate steps to intervene if symptoms worsen [5]. Self-care is the cornerstone of HF management. Patients who practice constant self-care have been shown to avert 30% of hospital admissions and more than half of the readmissions [6]. Despite its importance, several studies have reported gaps in patients’ knowledge and skills regarding HF self-care, with many patients finding HF self-care challenging, especially when transitioning from hospital care to home and community care [7,8]. Traditionally, hospital nurses have provided disease education with information on the value of medication adherence and lifestyle risk modification. Most of these education programs are done as part of discharge instructions and/or as part of inpatient education. However, the severity of symptoms during stressful hospital stays (eg, breathlessness and fatigue) precludes patients from being physically and psychologically ready to absorb any information. As a result, many patients feel underinformed when they return home [8].

We recently conducted a 3-arm stratified randomized controlled trial to evaluate the effectiveness of a newly developed, nurse-led, home-based psychosocial and educational intervention (home-based heart failure self-management program [HOM-HEMP]) for patients with HF in Singapore (ClinicalTrials.gov NCT03108235) [Jiang Y, unpublished data, 2021]. The development of the intervention and its detailed description have been published previously [9]. In brief, the HOM-HEMP intervention is a 6-week, multicomponent self-management program designed to promote HF self-care. It consisted of an HF patient education and self-care toolkit and three biweekly home visits by the research nurse. The toolkit included an HF education manual, a drinking cup marked with fluid volume, picture cards of common local foods and their associated sodium content, a weight scale, a weight monitoring calendar, and a pillbox with an alarm clock that reminded patients to take their medications (Multimedia Appendix 1). The toolkit provided cues (eg, a visual reminder of fluid intake and alarm reminder for medication adherence) of self-care activities in the context of daily life, whereas biweekly home visits provided an opportunity for the research nurse to interact with patients face-to-face, during which the research nurse also employed motivational interviewing to promote patient self-care engagement. In addition, a supplementary smartphone app was developed based on the study intervention (Multimedia Appendix 2), which was only available to participants in the experimental group B (one of the intervention arms). The smartphone app offered features such as educational content; customized scheduled medication and medical appointment reminders; weight, blood pressure, and symptom logs; and a chat room to communicate with the research nurse via text message. The research nurse reviewed the patient’s symptoms on the back end through a secured web portal.

The aims of the research project are to evaluate the effectiveness of the HOM-HEMP intervention in improving patients’ HF self-care, cardiac self-efficacy, psychological well-being, health-related quality of life, social support, and clinical outcomes. Consenting participants were randomly assigned to a control group, an experimental group A, and an experimental group B. All study participants received usual care provided by the study hospital. Participants in the experimental group A and experimental group B received usual care and the HOM-HEMP intervention. In addition, participants in the experimental group B received a supplemental smartphone app. The purpose of having 2 experimental groups was to explore the effects of the mobile health (mHealth) component on the study outcomes. The findings of this trial are reported elsewhere [Jiang Y, unpublished data, 2021].

### Objectives

This study details the process evaluation of the study intervention, which is part of the HOM-HEMP research project [9]. Process evaluations are recommended by the UK Medical Research Council guidance to provide additional information to understand how an intervention might or might not work in a specific context or whether the outcomes of the trial can be reproduced [10]. It is an important step in the evaluation of a complex intervention, as it allows researchers and practitioners to understand whether improvements in outcomes have resulted from the intended intervention process, whether the intervention causes poor outcomes, or even sometimes whether good outcomes are produced by the intervention that is actually less satisfactory [10].

To better understand participants’ views and experiences regarding the HOM-HEMP intervention provided to them, the Donabedian structure-process-outcome framework was employed in the process evaluation [11]. In his model, Donabedian proposed *structure*, *process*, and *outcome* of care as the key aspects of quality of care [12]. The framework recognizes that outcomes are associated with the relationships between the context and actions of patients and care providers and that understanding the implementation of an intervention requires not only what is delivered but also the mechanism through which the intervention is delivered [11]. In this study, *structure* refers to physical components of the intervention and the organization of the intervention, such as the duration and frequency of the intervention; *process* refers to the participants’ experiences of interactions and encounters during the study intervention; and *outcome* refers to changes in participants as a result of the intervention and/or interactions with the research nurse during the intervention.

The aim of this process evaluation is to explore participants’ views and experiences with regard to the HOM-HEMP intervention and to understand the mechanism by which the intervention had an impact.

## Methods

### Study Design and Participants

A descriptive, qualitative approach was used. One-to-one semistructured interviews were conducted to elicit participants’ responses to their overall experiences with the HOM-HEMP intervention. An interview guide was developed to guide the one-to-one semistructured interviews and to ensure uniform data collection across all participants. The questions in the interview guide were reviewed by the members of the researcher’s team, all of whom had experience in conducting qualitative research. The semistructured interview offers some flexibility that allows participants to voice out their views in a naturalistic narrative fashion while ensuring that all question areas were discussed with each participant [13]. Compared with group interviews, the one-to-one interview format provides a less threatening environment for the participants to share their personal views and/or describe their experiences [13].

Participants were recruited through a purposive sampling method from January 2019 to July 2019 at a tertiary public hospital in Singapore. Participants from the intervention arms with either positive or negative differences in any of the study outcomes were included in the process evaluation if they were willing to be audio recorded. The inclusion criteria for the HOM-HEMP research project were patients who were aged ≥21 years, had been clinically diagnosed with HF, were able to read and understand English or Chinese, owned and used a smartphone in their everyday lives, and were able to be followed up at home after discharge from the hospital. The exclusion criteria were patients who had unstable angina, resting tachycardia, or severe arterial hypertension; had a terminal illness other than HF that affects their self-care ability and/or completion of the study, for example, end-stage cancer; had a psychiatric condition or impaired cognitive functioning that affects their understanding of the study intervention; were bed- or wheelchair-bound affecting their mobility and self-care ability; and had no internet access at home. To ensure a diverse sample of participants, participants’ ethnicity, age, and socioeconomic status were taken into consideration when choosing the cases in this study to identify important patterns across variations [13].

### Sample Size

There is no established rule for sample size in qualitative evaluations. In this study, the sample size was determined based on information needs and the concept of data saturation [13]. Recruitment for the process evaluation interview stopped when no new information emerged and redundancy was achieved.

### Data Collection

At the end of the HOM-HEMP study, participants from either experimental group A or experimental group B were approached based on the purposive sampling criteria. Details regarding the interview process and objectives were explained to the participants. In addition, they were informed that the session would be audiotaped. Written informed consent was obtained from the participants before the interviews commenced. Interviews were arranged at a place convenient for the participant.

Qualitative data were obtained from individual, face-to-face, semistructured interviews. All interviews were conducted by a research nurse who delivered the study intervention. Interviews were conducted in either English or Chinese, depending on the participants’ language preference. Before the start of the interview, the research nurse reassured the participants that all the information they provided would be kept confidential and that the interview would be like a conversation with no right or wrong answers to the questions asked. Rapport was established by one or two warm-up questions before putting forward the topics on the interview guide. Open-ended questions were used to encourage participants to talk freely on the questions asked. If the participants’ family members were present during the process evaluation interview, their role was limited to helping to relay the point the participant was trying to make when the participant had difficulties in explaining his or her point of view clearly. All interviews were audio recorded and transcribed verbatim. The participant identifiers were omitted from the transcripts to maintain confidentiality. The transcripts were cross-checked by a second researcher to ensure accuracy.

### Ethical Considerations

Ethical approval was obtained from National Healthcare Group-Domain Specific Review Board (NHG DSRB ref: 2017/00249) before the commencement of the HOM-HEMP research project. Voluntary participation, confidentiality, the right to withdraw, and potential risks and benefits were explained. Informed consent was obtained from each participant before the study commenced. Participants were also reassured that declining to participate in the study would not lead to any penalties or any differences in treatment or care.

### Data Analysis

Descriptive data analysis was performed to profile the sample characteristics based on participants’ sociodemographic and clinical data. Frequencies and percentages were used to describe the categorical variables, such as gender, race, marital status, highest education level, employment status, types of housing, smoking status, the New York Heart Association functional classification, and the presence of comorbidities.

The thematic analysis approach was used to identify, analyze, and report the patterns (themes) within the data, as they are not reliant on any theoretical framework and provide sufficient flexibility for data analysis [14]. Specifically, the thematic analysis process involved five different phases, including familiarizing the data, generating initial codes, collating codes into potential themes, reviewing themes, and defining and naming those themes [14]. The transcripts were coded independently by the research nurse and a second researcher. The results were cross-checked for consistency and revised as required. The primary coding and collation of codes into themes were manually performed. To ensure that themes were formed coherently and accurately, the initial themes were reviewed and refined based on discussions with team members. Illustrative quotations were translated into English if they were in Chinese. Repeated words and grammatical errors were edited for ease of understanding, but no substantive changes were made. The translations were cross-checked by two researchers proficient in both English and Chinese.

### Trustworthiness

The trustworthiness of the qualitative approach in the process evaluation was established by ensuring the feasibility and adequacy of the semistructured interview guide in eliciting appropriate data to achieve the evaluation objectives. The questions in the interview guide were reviewed by members of the research team and the author’s supervisor, all of whom had experience in conducting qualitative research. In addition, to ensure the accuracy and authenticity of the data, the interviews were audio recorded and transcribed verbatim. The transcribed data and data analysis were cross-checked by two researchers, and thematic agreement was reached through discussion. These steps provided a complete and accurate description of the participants’ responses, thus ensuring the credibility of the process evaluation. The confirmability was to ensure that the themes were a true representation of the participants’ experiences and perceptions [13]. It was established by recording the participants’ quotations to support the results. Auditability was established by keeping records of the data analysis process, such as records of the evolution of findings and emergence of themes. Finally, transferability was established by providing a detailed research context to allow readers to relate to the conclusions drawn from this study.

## Results

### Sociodemographic Data and Clinical Characteristics of the Participants

A total of 11 participants were approached and invited to participate in the process evaluation interview, of which 6 were from experimental group A and 5 were from experimental group B. The average duration of the interviews was 9.24 minutes, ranging from 5 to 17 minutes. Of 11 participants, 7 (82%) were Chinese speaking. The descriptive statistics of the participants’ baseline sociodemographic data and clinical characteristics are presented in Table 1. Of the 11 participants, 7 (64%) were male and 4 (36%) were female. Their age ranged from 44 to 85 years (mean 64.45, SD 12.58 years). Of the 11 participants, 6 (55%) participants were still working full time or on a part-time basis. None of the participants drank alcohol, but 9% (1/11) of the participants had smoked in the past 4 weeks. From the 11 participants, 9 (82%) had coronary heart disease, 7 (64%) had diabetes, 6 (55%) had hypertension, and 7 (64%) had high cholesterol. 

**Table 1:** Sociodemographic data and clinical characteristics of the participants (N=11).

Variables			Values
Age (years), mean (SD)			64.45 (12.58)
**Gender, n (%)**			
	Male	7 (64)	
	Female	4 (36)	
**Race, n (%)**			
	Chinese	10 (91)	
	Indian	1 (9)	
**Marital status, n (%)**			
	Married	9 (82)	
	Unmarried, widowed, divorced, or separated	2 (18)	
**Highest education level, n (%)**			
	Primary school or no formal education	7 (64)	
	Tertiary (university, ITE^a^, polytechnic, or junior college)	4 (36)	
**Employment status, n (%)**			
	Working part time or full time	6 (55)	
	Not working or retired	5 (45)	
**Monthly household income, SGD^b^ (US $), n (%)**			
	<1000 (745)	6 (55)	
	1000-3000 (745-2236)	1 (9)	
	3001-5000 (2237-3727)	2 (18)	
	>5000 (3727)	2 (18)	
**Main caregiver, n (%)**			
	Family	8 (73)	
	Other (maid, friend, or alone)	3 (27)	
**Type of housing, n (%)**			
	2-3 rooms	3 (27)	
	4-5 rooms	6 (55)	
	Executive maisonette, private, or landed	2 (18)	
Ex-smoker, n (%)			2 (18)
Smoking (in the past 4 weeks), n (%)			1 (9)
Alcohol drinking, n (%)			0 (0)
**The New York Heart Association Functional Classification, n (%)**			
	I-II (mild to moderate)	1 (9)	
	III-IV (severe)	10 (91)	
Coronary heart disease, n (%)			9 (82)
Type 2 diabetes, n (%)			7 (64)
Hypertension, n (%)			6 (55)
High cholesterol, n (%)			7 (64)

^a^ITE: institute of technical education.

^b^SGD: Singapore dollars.

### Qualitative Findings

The findings are organized and presented based on the Donabedian framework. A total of six underlying themes representing participants’ overall experience and perceptions of the study intervention were identified. They were grouped under *intervention structure*, *intervention process,* and *intervention outcome* (Table 2). The emerging themes were manageability of the intervention, areas for improvement, benefits of visiting, personal accountability in self-care, empowered with knowledge and skills in self-care after the intervention, and increased self-efficacy in cardiac care.

**Table 2:** Themes and subthemes.

Themes		Subthemes
**Intervention structure**		
	1. Manageability of the intervention	Ease of learning Essential topics were covered Convenient to participate Different views on the use of technology
	2. Areas for improvement	Participants’ different preferences for frequency and duration of intervention Intervention format can be more flexible Some of the self-care tools did not match the participants’ usage habits To include topics related to chronic diseases
**Intervention process**		
	3. Benefits of visiting	Allow patients to better express themselves Preferred personalized advice over information from other resources Felt nurses can better understand patients’ problems Reassurance for family members Worries and burden are being addressed
	4. Personal accountability in self-care	You have to help yourself first Self-discipline is required
**Intervention outcomes**		
	5. Empowered with knowledge and skills in self-care after the intervention	Monitoring inculcated in daily life Able to sustain effort after intervention
	6. Increase self-efficacy in cardiac care	Increased initiative Improved self-awareness of health status Determination to change a certain lifestyle

#### Intervention Structure

##### Theme 1: Manageability of the Intervention

Most participants reported that overall, the intervention was easy to manage with no added burden. It covered all the essential topics that participants wanted to know with an adequate amount of information. The most helpful aspects of the intervention included coaching on weight monitoring and controlling fluid and salt intake. One participant mentioned that it was important that the intervention be as simple as possible so that it would help them apply what they had learned easily:

...[the programme] is really very good, because I have learnt a lot of things that I did not know last time, like how to read the sodium amount in the food label...

P2, aged 46 years

...[the most helpful aspects] are weight monitoring, coz it tells me if I have water retention...also controlling my water intake and diet...I try to keep my water intake to 1L/day, and eat less oily and salty food...

P6, aged 76 years

I think the best is simple and useful...yes...because we need to work, if it is too complex, we feel very troublesome. So, I think the simpler the better...And also, we are not that young now, sometimes we cannot remember so many things.

P9, aged 59 years

In addition, home visits reduced the travel needs of the participants. Convenience of participation through home visits encouraged participants to participate in the study. For some participants, the flexibility of scheduled home visits allowed them to plan ahead between different commitments:

Look, instead of you asking patients come over [to the hospital], you are visiting them now. So it is more convenient for the patients as well. Rather than you ask them to come to the hospital, you are meeting them halfway at their premises. It is a good thing and I think a lot of patients will like it. It is easier. If you ask me to go down to the hospital I might be like “ah, cannot today, tomorrow cannot.” I may want to push the appointment for the later dates.

P8, aged 58 years

I think it is good because you will call me to confirm home visit appointment before you come, and if I cannot make it, I can also give you a call. This is very good...

P6, aged 76 years

In terms of the mHealth component of the study intervention, participants had different views on the usability and acceptability of the technology. Some participants reported that employing the smartphone app in health monitoring increased their awareness of their personal health condition and found it convenient to record and monitor their condition via the app. Importantly, having the nurse and other health care professionals accessing and monitoring the participants’ conditions at the backend was especially useful in motivating them to use the app. However, older patients needed to rely on their helper to monitor their health data because of difficulties in using a smartphone:

...initially, it was not easy to use, there were some errors in [Chinese] translation...subsequently, this problem has been fixed, we can understand [the meaning], and it becomes very easy to use...app is preferred over paper and pen, because you just need to press a few buttons, if [you] record on paper, you need to first look for a pen, then write it down...

P6, aged 76 years

...I prefer the app...I really appreciated that. It gives you a purpose that you want to do it every morning to key in whatever information. So that it can be shared all over in the hospital. They are also looking at it. And it’s so easy. I think the app is user friendly. No problem.

P8, aged 58 years

We don’t know how to use smartphone, it is difficult for our age, so I have to ask my helper to key in the record.

P3, aged 83 years

##### Theme 2: Areas for Improvement

Participants also made suggestions on intervention frequency, duration, and content coverage for future improvement. A male participant believed that the interval (ie, every 2 weeks) between home visits was too short and the duration of the intervention program (ie, 6 weeks) was too long. In contrast, female participants felt that the frequency and duration of home visits could be increased. The participants also preferred that the disease education and self-care content provided by the research nurse could cover other topics related to chronic disease management and not just HF. Furthermore, one participant wanted the research nurse to relay his information to the doctors so that he did not need to repeat himself during the doctor’s consultation, as consultations are usually very short:

Personally, I feel that the home visit interval is too close, may be one or two months will be ok...yes, both are ok, if home visit interval increased, give a telephone call in between home visits is also fine...

P9, aged 59 years

I hope that your programme duration won’t be so short, it will be good if you can visit the patient two to three times a month. Or perhaps, visit more frequently at the start, and if patient is stable, can slowly [reduce the frequency]...but will continue to see them...

P2, aged 46 years

..., but now you can only teach us about heart problems...Sometimes it’s inevitable that there will be other diseases, although I hope I won’t be so unlucky as to have other diseases after this heart problem...But things like high blood pressure, high cholesterol, and high blood sugar are common and can be discussed...

P10, aged 58 years

I hope that whatever you saw during home visit, [you] can let my doctor know too...my problems...because doctor’s consultation is very short, only have 10 minutes...but during home visit, I can tell you more...

P3, aged 83 years

Finally, not all participants used all the tools in the HF self-care toolkit provided by the research team for self-care. Participants felt that some of these tools did not fit their usage habits. The size of the pillbox in the self-care toolkit was too small, and the weight monitoring calendar was too complex to use. Participants preferred to record their weight in their own way, rather than following it on weekly pages, as instructed:

...among all the things you gave me, I did not use the table [referring to the weight monitoring calendar], and the pillbox is too small, not big enough to put all my medication in, you should have given me a bigger one...

P2, aged 46 years

the pillbox in the self-care toolkit is less useful, because if I put my medication inside, I cannot see the medication instructions and the name of the medication, also the size of the box is too small...

P6, aged 76 years

Basically, everything is ok, but...except for the weight record calendar, it is too complicated, I only need to keep my own record...

P9, aged 59 years

#### Intervention Process

##### Theme 3: Benefits of Visiting

Participants reported that they preferred a nurse to visit them for a variety of reasons, including better clarity and understanding with face-to-face interaction for both the nurse and participant, enabling increased depth of discussion and the family being reassured with an understanding of their conditions, and that they were well taken care of. Participants also felt that their thoughts and existing problems could be better understood during home visits. Older adult patients stated receiving too much information from the news and internet nowadays, which can confuse them sometimes. However, through face-to-face interactions, the nurse can probe and discover minor problems encountered by the patient that might otherwise go unnoticed:

I find it hard to communicate clearly through phone, it is better to talk face-to-face, it is much clearer in this way...[P6, aged 76 years]

I preferred face-to-face interaction, because sometimes, we wanted to clarify something...I mean when you are trying to describe some problems, it is very hard to talk over the phone. Therefore, I think home visit and face-to-face interaction are much better.

P9, aged 59 years

I think it is very important to talk face-to-face, because you received too much conflicting information from computer...the newspaper said coffee is good for the heart yesterday but now it said [coffee] is not good [for the heart], so you don’t know which information is correct...and become very confused...

P3, aged 83 years

In addition, some participants, especially those who were staying alone or only with their spouse who is an older adult, identified that the relational component in the intervention process made a difference to them. Perceived caring presence can be as simple as individual attention, understanding of their concerns, or comfortable interactions. During face-to-face home visits, a participant felt that the ability to voice out his problems to a nurse who is knowledgeable about HF was helpful in unloading his burden:

...I would love to see you coming...otherwise the house will be so quiet...

P4, aged 58 years

Yes, yes I do. And I really appreciate that because you took your time to come to my home, be there. It wasn’t a very formal meeting. It’s like talking to friends. So, it is good...we can just talk, not just about medical advice. We can sit down and talk, and have a good conversation.

P8, aged 58 years

When you are talking to somebody, you are putting whatever your problem or whatever elements, you are just voicing out. Just like some help to unload the burden that you’ve been carrying along. And talking to you, whoever the interviewer is, they know about the subject. So, it helps, you know they might understand. But talking to family member is just the basic, I don’t know. They may not understand. So, it is very much helpful.

P8, aged 58 years

##### Theme 4: Personal Accountability in Self-Care

During the intervention process, participants recognized their own personal responsibility in HF self-care and felt that self-discipline was required to benefit from the study intervention:

actually, it’s very helpful. At the same time when people try to help you, you yourself must help you. You have to be disciplined as well. That means whatever the doctor or the nurse advises you, you have to follow it. But at the same time, you have to gauge all these yourself, to see whether the conditions suit you or not. But most important thing is to discipline yourself. If you don’t discipline yourself, nobody can help you.

P11, aged 57 years

#### Intervention Outcomes

##### Theme 5: Empowered With Knowledge and Skills in Self-Care After the Intervention

A couple of participants mentioned that they will continue to use the intervention materials, such as the measuring cup and weighing scale, to monitor HF even after the intervention ended. They expressed sustained confidence in incorporating changes after the intervention, as regular weight monitoring and control of diet have been inculcated in daily life:

I feel my physical health and mood have improved...I used to be less energetic, but now I feel my energy level goes up...and I feel more confident [to manage my condition] after someone taught me these methods...yes, yes, I will definitely continue to maintain healthy diet, control my water intake...will follow the instructions...

P5, aged 69 years

last time, I did not know soup can be salty, so I keep drinking soup and I thought that was good...but now I cut it down totally...when I go to supermarket, I will also look at the food label and pay attention to the sodium content...in the past, I only look at place of production and nothing else...

P2, aged 46 years

...I have been following the instructions on the HF manual...I have done that...see I used the measuring cup you gave me to make tea, with no sugar...I like to use the [measuring] cup, I have been using it and I also keep track of my weight every day, it was 65 point something in the past, now is 62.5, 63.8 and then no further increase...I lost some weights and [feel] less tired...I prefer to keep my current weight, and I am happy about it.

P4, aged 58 years

##### Theme 6: Increased Self-Efficacy in Cardiac Care

Participants reported that one of the biggest changes after the study intervention was the ease of control of symptoms due to a better understanding of the HF condition. They felt that they were gaining more control in managing their HF symptoms. A few of them showed increased initiative to control their symptoms. For example, a participant would control his activity level to reduce dyspnea. Another participant reported that he kept track of his weight to better understand his current health and adjust his diet accordingly. When necessary, they would take the initiative to act quickly:

I can control my condition more easily because I have more knowledge about how my condition is right now...I am quite confident...

P11, aged 57 years

I walk, but not very fast...and avoid strenuous exercises. I still practise tai Chi for a while, but not too long...

P3, aged 83 years

...I think after quitting smoking, finally I am getting on my way. And there is a way...

P8, aged 58 years

it is better to record down as it helps me to better understand my current health condition...and I started to pay more attention to my diet, so as not to aggravate my condition...I started to control, so I cook myself...

P10, aged 58 years

## Discussion

### Principal Findings

A total of six themes (manageability of intervention, areas for improvement, benefits of visiting, personal accountability in self-care, empowered with knowledge and skills in self-care after the intervention, and increased self-efficacy in cardiac care) emerged from the process evaluation and summarized participants’ experiences and perspectives after receiving the HOM-HEMP study intervention. Overall, participants expressed more advantages than disadvantages of the study intervention. In addition, participants appeared to be more attuned to the process of the intervention and the experience of interacting with the research nurse than to the content and form of the intervention. Thus, the strengths they expressed were more focused on the intervention process than on the intervention components. This was also reflected in the fact that although the HF self-care toolkit provided by the research team was not perfect, this did not prevent them from using other methods for self-care.

In this study, home visits remained to be the preferred method of engagement for most patients. Although home visits may be perceived as resource intensive, they have intangible values. Face-to-face interactions allowed for more authentic and in-depth exchanges between the participants and the research nurse. Participants felt that they were being listened to, rather than being rushed to end the conversation, and gave the nurse an opportunity to probe and discover problems that might otherwise have gone unnoticed. In addition, human touch and care remain to be important in the healing process. Personal encounters help foster therapeutic nurse-patient relationships that will further enhance their recovery process [15]. This was further demonstrated when some participants reported that the relational component and the perceived caring presence in the intervention process had an impact on them. This finding is consistent with the results of a previous review on the effective mechanisms of disease management interventions in HF [16]. In their review, Clark et al [16] found that studies with effective health professional supports were those with *sufficient* consultation time, who incorporated patient goals into care, providing rapid feedback, and who had a consolidated patient-professional relationship. Conversely, interventions are less effective when health care providers focus too much on mere information giving or prioritizing treatment goals over the patient’s own goals [16].

In this study, participants reported that they became more confident in self-care as a result of increased knowledge and understanding of symptom monitoring and interpretation. Effective intervention promoted the understanding of many complex links between HF symptoms and self-care tasks [17]. Although a plethora of accessible health information exists on the internet, in newspapers, and on instant messaging tools such as WhatsApp, a considerable proportion of patients are left with either an incomplete or an incorrect understanding of their condition, as they are not equipped to discern this vast amount of information. Even when they have the right information, guidance in translating their knowledge and skills into pragmatic actions is needed. As such, the consultative role of the nurse has evolved from simply providing health information to helping patients sift through and ascertain the credibility and authority of the informational sources, identify knowledge gaps, dispel confusion, and provide personalized advice. Participants seemed to particularly value this kind of support from health care professionals. This can be seen in the participants who expressed the wish that the research nurse could have also included education topics on other chronic disease management besides HF. Individualized interventions that are responsive to individual learning needs and preferences, with personalized reinforcement of specific salient information, have been shown to be effective in promoting self-care behavior change [17-19].

Although mHealth and telemedicine have great potential to overcome many barriers to health care delivery [19], especially in the context of the current COVID-19 pandemic [20], participants had different views on the use of technology in the study. Some believed that employing a smartphone app in health monitoring was not only convenient but also increased their awareness of personal health. One of the main motivations for participants to use the app was that they knew that their recorded data on the app would be monitored backend by the research nurse. Athilingam et al [21] reported that mHealth apps have the potential to improve self-care by providing patients with real-time access to health care providers. Similar observations were found in an unpublished local study in which postmyocardial infarction patients highlighted that the greatest advantage of telemedicine interventions was the ability to have direct access to their health care providers [22]. Although participants commended the many benefits of using a smartphone app in their health monitoring, older patients needed to rely on their caregivers to monitor their health data because of the difficulties in using a smartphone. Past research has reported that the efficacy of mobile phone–based telemonitoring in improving HF outcomes may depend on the characteristics of the patient population [23,24]. In this regard, further trials are needed to explore the effectiveness of mHealth apps in defined patient subgroups.

In this process evaluation, participants provided a lot of valuable feedback to maximize the study intervention in future studies, particularly on the structure of the intervention. First, interventions should be kept simple and manageable so as not to overwhelm or intimidate the user. Second, there should be some flexibility in the implementation of study interventions. Depending on the patient’s progress, the interval between home visits can be extended or shortened. Long intervals between home visits can be augmented by telephone calls to check on the patient. In addition, one interviewee suggested that the intervention duration be extended as needed. Finally, although the HF self-care toolkit was designed to help patients establish a systematic self-care process and provide some tangible help in integrating self-care into their daily lives, not all of the items in the toolkit were user-friendly. Consequently, some participants did not like to follow up on tracking their weight trends and managing their medication in the way they were told.

### Limitations

It is inevitable that this study had limitations that may affect the transferability of the research findings. First, as the research nurse for this study delivered both the study intervention and conducted the interviews for the process evaluation, this may have led to bias in the collection of participant feedback because of an established rapport between the participants and the research nurse. However, the development of rapport is not only a limitation but can also be seen as a strength. When participants are familiar with the interviewer, they will be more open to speaking and sharing their genuine opinions. Moreover, as the interviews were semistructured, in addition to discussing the parts of the intervention that were found to be helpful, we made a point of exploring with participants where the research interventions were least helpful and where future improvements were needed. These measures facilitated a relatively balanced discussion with participants. Second, some participants were very reticent during the interview process; even after much probing, they just answered *everything is fine* or repeated what they had already mentioned. As a result, some of the interviews were very short, which may have affected the authenticity of the data.

### Conclusions

Despite these limitations, the findings of the process evaluation provided additional information about participants’ perceptions and experiences with the HOM-HEMP intervention. Although a home visit may be perceived as resource intensive, it remains to be the preferred way of engagement for most patients. During the intervention, the nurse played an active and essential role in the individual’s healing process, further demonstrating that not only does the value of nursing come from the content of the intervention but that interactions with the patient can be an important empowering process for self-care behavior changes. These findings shed light on why and how the intervention worked and areas for improvement in future studies. It allows researchers and practitioners to better understand the mechanism by which an intervention generates impact. Therefore, future intervention programs can leverage these mechanisms and adapt them to different contexts for better intervention effectiveness.

## Appendix

Multimedia Appendix 1 The heart failure education and self-care toolkit.

Multimedia Appendix 2 Heart failure supplementary smartphone app.

## Abbreviations

HF: heart failure
HOM-HEMP: home-based heart failure self-management program
mHealth: mobile health
