# Supplementary figures and images for: Patients’ Experiences of a Nurse-Led, Home-Based Heart Failure Self-management Program: Findings From a Qualitative Process Evaluation
Source: J Med Internet Res. 2021 Apr 27;23(4):e28216. doi: 10.2196/28216 (PMC8114165; doi:10.2196/28216)

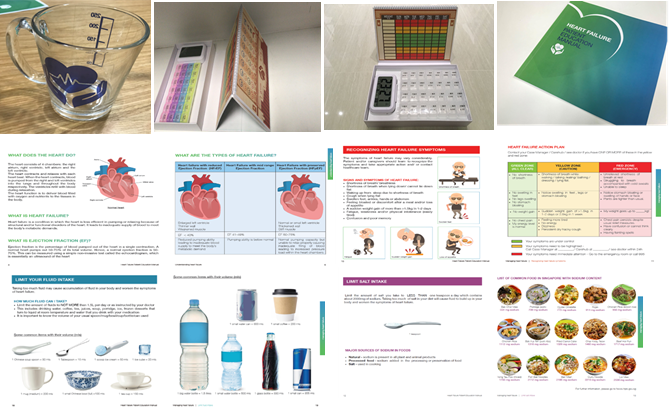

Supplement: Multimedia Appendix 1 [file jmir_v23i4e28216_app1.png]

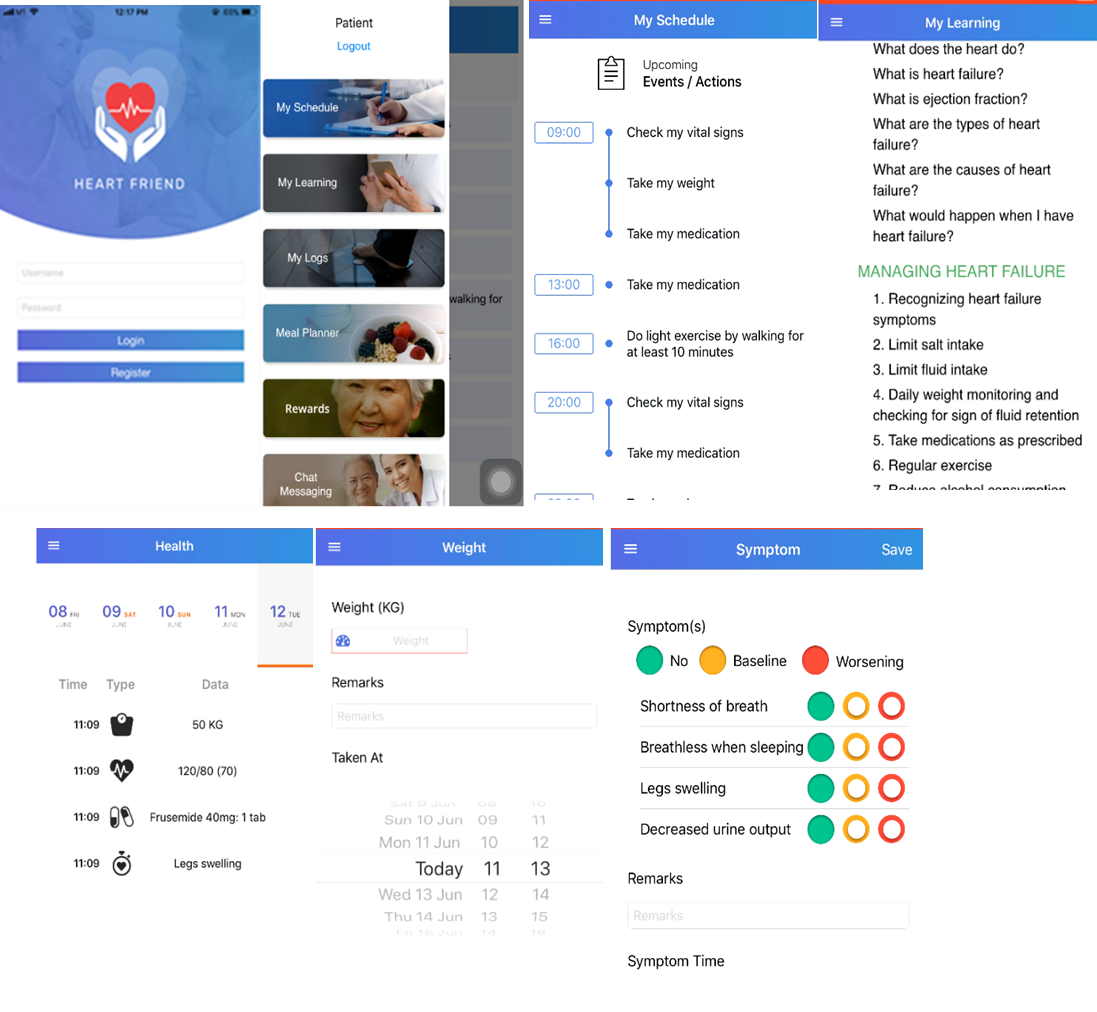

Supplement: Multimedia Appendix 2 [file jmir_v23i4e28216_app2.png]
